# Knowledge, beliefs and perceptions of religious leaders on modern contraceptive use in Burkina Faso: a qualitative study

**DOI:** 10.11604/pamj.2021.39.216.27082

**Published:** 2021-07-28

**Authors:** Abibata Barro, Noufou Gustave Nana, Dieudonné Soubeiga, Nestor Bationo, Yacouba Pafadnam, Hermann Pilabre, Patrice Alain Ngangue

**Affiliations:** 1 Institut de Formation et de Recherche Interdisciplinaires en Sciences de la Santé et de l’Education, Ouagadougou, Burkina Faso,; 2 Faculty of Medicine, Department of Family Medicine, McGill University, Montreal, Canada

**Keywords:** Religious leaders, contraception, qualitative research, Burkina Faso

## Abstract

**Introduction:**

in Burkina Faso, despite the strategies implemented to increase the use of contraceptives, the prevalence rate of modern contraceptives remains low. Religion is an important part of the socio-cultural fabric of many communities. Besides, religious leaders play an essential role in adopting and using contraceptive methods to support family health. The study objective was to explore the knowledge, beliefs and perceptions of religious leaders about modern contraceptives among women of childbearing age.

**Methods:**

data were collected in September 2018 from twenty-one religious' leaders of the urban municipality of Dori. Study participants were selected based on reasoned sampling with maximum variation (sex, religion, age, residence and level of education). We conducted semi-structured individual interviews, non-participant observations and documentary review.

**Results:**

religious leaders have a good knowledge of modern contraceptive methods, but they prefer traditional contraceptive methods and abstinence. They consider modern contraception as abortion and female sterilization and emphasize birth spacing. Furthermore, religious leaders lack training on contraception and have no real links and exchanges with sexual and reproductive health services. As a result, their assessment of the quality of these services is very mixed.

**Conclusion:**

religious leaders play a crucial role in improving modern contraceptive methods in Burkina Faso. Close collaboration with family planning services should, at all times, be maintained. The implementation of training and educational activities for religious leaders could help raise modern contraceptive use in Burkina Faso.

## Introduction

Family planning is part of the United Nations sustainable development goals (SDGs), which call for universal access to sexual and reproductive health care services, including family planning, information and education, and the integration of reproductive health into national strategies and programmes. Goal 3.7 “health and well-being for all by 2030”, aims to improve maternal, newborn and child morbidity and mortality among that population [1]. Like other countries in sub-Saharan Africa (ASS), Burkina Faso has a maternal mortality ratio (MMR) of 330 per 100,000 live births in 2018 [2]. This country is characterised by a low rate of modern contraceptive methods, with 23.3% unmet need for family planning and a contraceptive prevalence rate of 32.5% [2]. Studies on unwanted pregnancies and abortions report that three out of ten pregnancies are undesirable and one out of three unintended pregnancies end in abortion [3].

In addition, unplanned births cause enormous health and development problems in sub-Saharan Africa. Indeed, children and women are predisposed to massive health problems in high-fertility families (high child and maternal morbidity and mortality) [4]. As a result, the government of Burkina Faso has committed to increasing modern contraceptive prevalence through several strategies such as the community-based distribution of contraceptives, implementation of a strategic plan to secure reproductive health products and a national plan to revive family planning [5]. In addition to implementing the national action plan to accelerate family planning 2017-2020, local authorities and religious leaders are asked to develop outreach activities to raise awareness in their communities [6].

Religious beliefs, fertility issues, contraceptive adoption and abortion, can differ considerably among protestant, catholic, Muslim and traditionalists [7]. For example, abortion is prohibited in all these religions. However, most schools of thought may allow early and therapeutic abortion when the mother's life is in danger [7]. The doctrine of the Catholic Church allows only natural methods of contraception [7]. Many religious leaders have opposite convictions to the use of modern contraceptive methods [7]. As a result, they can significantly influence the demand for family planning [7]. That is why religious leaders are called upon to promote modern contraception and reduce social inequalities that affect women in particular [8].

Although numerous studies have assessed the role of religious beliefs in contraceptive adoption and family planning [9], there are data gaps regarding the role of religious leaders in the adoption and use of contraceptive methods. Therefore, our study aims to contribute to promoting women's reproductive health in Burkina Faso by exploring the knowledge, beliefs, and perceptions of religious leaders about the contraceptive practices of women of reproductive age.

## Methods

**Study design and setting:** this study was conducted in the urban municipality of Dori. It was a descriptive qualitative study. Sandelowski states that qualitative descriptive research is an ideal method for describing people's experiences and perceptions in a given situation [10]. We followed the consolidated criteria for reporting qualitative studies (COREQ) guidelines for reporting qualitative research (Annex 1).

**Participants:** the participants were religious leaders from the Dori urban municipality. Purposive sampling with maximum variation resulted in a sample of 21 participants. The eligible religious leaders had to meet the following criteria: 1) be a muslim, roman catholic, protestant or animist spiritual leader officiating in places of worship in Dori urban municipality; 2) give informed consent to participate in the study; 3) reside in Dori urban municipality and; 4) be at least 18 years of age.

**Recruitment:** to ensure that all participants were represented, the executive secretary of the Union Fraternelle des Croyants (UFC) of Dori helped identify the different religious leaders spread across the five sectors of the urban municipality of the urban city Dori. Then letters of invitation were sent to all religious leaders in the urban commune of Dori. During this first meeting at UFC headquarters, the study plan and procedure were explained to the participants. Furthermore, the phone contacts of those interested in participating in the study were recorded. Finally, they were called to set the place, day and time for the meeting for the individual interview.

**Data collection:** data were collected in September 2018. A semi-structured individual interview guide was developed iteratively. The individual semi-structured interview was chosen because this technique “combines a non-directive attitude which favours the exploration of thought in a climate of confidence and a directive project to obtain information on points defined in advance” [11]. The interview guide made it possible to collect, in addition to the variables on the socio-demographic characteristics of religious leaders (residence, age, sex, level of education and religion), the knowledge of religious leaders on modern contraception; the use of information on modern contraception by religious leaders in religious activities; existing relationships between religious leaders and reproductive health services. An informed consent form was submitted for signature to each participant before each interview. Semi-structured interviews took place in the home or the workplace of each religious leader. They were recorded using a dictaphone. Each interview lasted approximately 20 to 35 minutes. The non-participant observation was also used to collect data on the behaviours and practices of religious leaders while staying at the scene. It consisted of taking field notes in a logbook. Finally, we carried out a documentary review which enabled us to identify, consult and use administrative documents relating to the roles and responsibilities of religious leaders in the exercise of their functions. This information was collected using a collection sheet developed for this purpose.

**Data analysis:** a thematic analysis of the collected data [12] using QDA miner software from Provalis (version 4.1.3) was carried out [13]. The analysis began simultaneously as the data collection to be “dynamic, constantly progressing, constantly fed by the work in the field” [14]. First of all, the audio recordings were transcribed in verbatim form. Next, the field notes and the content of the collection sheets were reviewed, corrected and recorded in a Microsoft Word file. All the material was read several times to absorb the different data. Subsequently, the data were inductively coded [15]. The initial themes were grouped under central themes. Similar themes were merged and prioritised [12]. To ensure the validity of the results and confirm the consistency of our interpretations, the preliminary analysis was presented to key informants.

**Ethical considerations:** Sahel Regional Health Directorate approved the study (survey authorisation N° 2018-084/MS/RSHL/DRS of 12^th^June 2018). The study protocol was validated and approved by the Burkina Faso Health Research Ethics Committee (N° 2018-7-095 du 19^th^ July 2018). All participants gave their written informed consent.

## Results

**Socio-demographic characteristics of religious leaders:** the sampling for this study consisted of 21 religious leaders working in the urban municipality of Dori who were interviewed. Table 1 shows the socio-demographic characteristics of the interview participants. The sample consisted of nine women and twelve men. Their ages ranged from 37 to 78 years old. Three of the participants had primary education, ten of the participants had secondary education and eight had a high level of education. In addition, nine participants were Muslims; seven were Roman Catholics and five protestants, all spread across the five sectors of the Dori urban municipality (Table 1).

**Table 1:** socio-demographic characteristics of the study population

Number	Code	Age	Gender	Education	Religion	Area/neighborhood of origin
1	LEAD_01	53	M	Higher	Catholic	Sector 05
2	LEAD_02	78	M	Secondary	Muslim	Sector 03
3	LEAD_03	42	F	Higher	Catholic	Sector 05
4	LEAD_04	38	F	Secondary	Catholic	Sector 04
5	LEAD_05	51	M	Higher	Muslim	Sector 02
6	LEAD_06	37	F	Secondary	Muslim	Sector 02
7	LEAD_07	47	M	Higher	Catholic	Sector 01
8	LEAD_08	49	F	Secondary	Muslim	Sector 02
9	LEAD_09	50	F	Secondary	Protestant	Sector 05
10	LEAD_10	56	M	Secondary	Protestant	Sector 01
11	LEAD_11	62	M	Higher	Catholic	Sector 05
12	LEAD_12	51	F	Secondary	Catholic	Sector 03
13	LEAD_13	45	F	Secondary	Protestant	Sector 01
14	LEAD_14	45	M	Secondary	Muslim	Sector 04
15	LEAD_15	40	F	Secondary	Muslim	Sector 03
16	LEAD_16	41	M	Higher	Protestant	Sector 01
17	LEAD_17	71	M	Primary	Protestant	Sector 01
18	LEAD_18	52	M	Higher	Catholic	Sector 02
19	LEAD_19	62	M	Primary	Muslim	Sector 05
20	LEAD_20	44	F	Higher	Protestant	Sector 01
21	LEAD_21	58	M	Primary	Muslim	Sector 04

M: male; F: female

**Knowledge of religious leaders:** for the participants, birth control was seen as a necessity. They all maintained no religious contraindications to birth control, as the following discussion shows: *"absolutely! It is important to control the births because birth control did not just start now"* (LEAD_01). We noted that most participants had a good knowledge of the most common modern contraceptive methods through the interviews. The pill, the intrauterine device (IUD) and the implant were mentioned most often: *"I know that we are talking about the pill, it is not new. They also talk about the injection; it is more recent. We are talking about the IUD! Norplant, I have seen people do it"* (LEAD_02).

From the perspective of most of the women leaders interviewed, modern contraceptives help women to space births. However, while they believe in the safety and efficacy of some methods, such as implants, they categorically reject the IUDs and prefer natural methods. For example, this participant says: *"with Norplant, I am not afraid to have a child right away [...]. I rest at least three years before having another one"* (LEAD_09). On the other hand, another participant says: *"of course I do not participate in the promotion of these methods! [Laughter], I promote the natural method. [...] this IUD consists of interrupting pregnancies..."*. Moreover, she goes on to say: *"[...] some people have become sterile because of the use of these methods. Some are no longer healthy because of this; they will end up getting cancer"* (LEAD_03).

On the other hand, the verbatim analysis shows that religious leaders disapprove of modern contraception because they equate it with a birth limitation. According to the participants, women's modern contraceptives are an obstacle to procreation because they are the generosity and providence of the Creator. On this subject, one of the participants stated: *"I disapprove of contraceptive methods. The prophet Mohamed said He would like his entire community to be the largest of the communities that have gone before us. A child is a gift of God that could never be refused"* (LEAD_08).

In general, the interviews with participants revealed a relatively good understanding of the rationale for capturing the demographic dividend (change in the age structure of a population, to bring about economic development) as reported by this participant: *"the demographic dividend consists quite simply in promoting birth control policies that make it possible to ensure that the available resources are managed in a consistent way with the populations of the country to be managed. This is in line with policies that encourage family planning in the context of countries in the sub-region, such as Burkina Faso"* (LEAD_05). However, some participants do not share this point of view: *"it must be proven that by reducing births, we can achieve economic growth. I do not believe this! Because China and Japan are highly populated but at the same time well-developed countries"* (LEAD_03).

**Raising awareness during religious activities:** depending on their level of education and religion, each leader brings their message to their activities. Leaders with higher levels of education report spreading messages about birth spacing at religious activities without encouraging the use of modern contraceptives. The words of two of the participants are revealing in this regard: *"I chaired an organisation, a network of Islamic associations on population and development. We had an awareness-raising programme focused on family planning and reproductive health issues. However, the awareness-raising advice did not focus exclusively on the issues of modern contraceptive methods"* (LEAD_05). *"We haven't raised awareness yet, but there is an organisation [...] Not to talk about all these methods but about the vision of the church. Man has a conscience, and man does not own his life, man receives everything from God [...]"* (LEAD_07).

As for the secondary level, participants are adamant and do not spread any messages on reproductive health during the activities, sometimes for reasons more personal than religious reasons. Again, the words of this participant illustrate this well: *"I am not talking about that! No, I am not going to do an awareness programme for that! Because I am not the right role model. I am not the right role model because I have many children, and then I have had many women!"* (LEAD_02).

**Relationships between religious leaders and reproductive health services:** the religious leaders interviewed stressed that they are not sufficiently involved in reproductive health activities. They rarely have opportunities to meet at both the national and regional level, but only locally. One participant said: *"I have not yet participated in a national or Dori meeting on family planning. They usually send a letter; saying that something is going to happen"* (LEAD_02).

Most of the participants expressed some dissatisfaction with the quality of family planning services, particularly about the reception, the attitude of the health professionals, the equipment and resources of family planning services. The words of three participants illustrate this perfectly: *"I can happen that the healthcare staff members are often not up to the scratch on issues of reception and sociability [...]. Others are there for the money! You can sense that they are there for the name"* (LEAD_10). *"I know that our services are making some great efforts, but I also know that the set is not very well provided, but it is in improving little by little"* (LEAD_01). *"These people are trying to do what they can to fulfil their mission. However, the problem lies in terms of insufficient human, material and financial resources. Furthermore, of course, when you do not have all the resources, you need you cannot achieve the results you want"* (LEAD_05). A summary of the main themes and determining factors is presented in Table 2.

**Table 2:** involvement of religious leaders in the promotion of the modern contraceptive practice

Main themes	Determining factors
Knowledge of religious leaders	On birth control
	Modern contraceptive methods
	On capturing the demographic dividend
	Religion and MCP
Use of the information on modern contraceptive methods in activities	Message during religious activities
	Religious curriculum
Relationship with RH health services	Effectiveness
	Appreciation of RH services
	Adoption of modern contraception

MCP: modern contraceptive practice; RH: reproductive health

## Discussion

This study explores the knowledge, beliefs and perceptions of the religious leaders about modern contraceptive practices for women of reproductive age. The findings were grouped into three main themes: religious leaders' knowledge of modern contraceptive methods, their training on modern contraception issues and their relationships with family planning services.

The results showed that the religious leaders interviewed have a good knowledge of modern contraceptive methods. The majority cited the condom, the pill, the IUD and the implant. These results are consistent with those of the study conducted in Burkina Faso, which showed that the four best-known methods are the condom, the pill, the IUD and the norplant [16]. Exposure of the general population, including religious leaders, to awareness campaigns on family planning (FP) and human immuno-deficiency virus (HIV) prevention through the media and/or communication tools such as the internet, telephones, or friends could explain this state of affairs [8,17]. Since these leaders need to enlighten their communities, this knowledge is an asset. However, the majority do not know the definitive methods of modern contraception. This situation would have explained that the latter is the subject of very few mass communications because they are considered specialised (doctors, midwives, nurses etc.) [18,19]. Despite reasonably good knowledge of modern contraceptive methods and the need to space birth, religious leaders do not adhere to and not recommend modern methods. They believe that modern contraceptive methods are morally unacceptable. They consider that family planning services do not respect the freedom of the spouses, such as the right to bear children. Other authors have made similar findings that the use of hormonal contraceptives remains problematic in rural areas [20,21] and that fertility preference underlies the social value of procreation as an obstacle to modern contraception methods [22].

Consequently, a pro-natal view is put forward. The pressure exerted by religious leaders on women is extreme, and modern contraception remains low [8,23,24]. The resistance of religious leaders to promote modern contraceptives is a fundamental educational problem that requires a more pedagogical approach [25]. This could explain the wide gap between the religious leaders' levels of knowledge about modern contraception and women's use of contraceptive methods.

The religious education curriculum does not include information on modern methods of contraception. These results are similar to those found in the study by Pinter *et al*. [26], which shows that the Holy Koran does not inform directly about family planning. However, in different suras, the Qur'an refers to the protection of the family. In contrast, Shrestha *et al*. [27] find that the Qur'an does not mention infanticide, leaving room for religious leaders and other believers to make multiple interpretations of the status of family planning, including modern contraception methods. In the Qur'an, it says: “Allah wants the easy things for you. He does not want the hard way for you”. Islam is a simple religion of ease, where there should not be either rigidity or excesses that would only serve humans [26]. It was also explained that religious leaders are firm in prohibiting most contraceptive methods and any recourse to abortion, as the Bible points out in the book of Genesis. The function of the family is to reproduce new souls while remaining in natural fertility, which corroborates our findings [28]. Some participants reported that contraceptives lead to sexual debauchery and infidelity among women and girls. Studies abound in the same direction, justifying that losing control over women's fertility is the fear of losing control over their sexuality that worries [29,30]. These interpretations of modern, which ultimately dictate a point of view and direction for action, are partly based on ignorance of the programs [24,26].

The relationship that religious leaders have with family planning services does not allow them to improve the quality of these services. A study has shown that despite the government's structures and means with international aid, the lack of communication with beneficiaries is a handicap to their use [24]. A religious leader emphasised that relations with family planning services result in a gap between the health structures and the populations they are supposed to work with. The social mobilisation around family planning does not match the heath authorities' ambitions about religious leaders. On this point, Hadiza argues that religious leaders are not fully involved in the actions of the modern contraception sector [31]. Indeed, information must also pass through religious leaders to prepare them psychologically and materially at all health system levels. The health system only provides space for services and actors other than technical ones; therefore, there is no place and role for other actors such as community leaders. We are in a process that has begun, and we need to be somewhat patient and suggest that we strengthen the dynamic. Thus, to achieve the promotion of family planning among women, the contribution of religious leaders becomes fundamental. They must have access to reliable and complete information they can transmit to fight the reluctance of certain religious circles [8]. Communities and their leaders, spiritual leaders or other influential persons must be involved to ensure the quality and continuity of services and monitor the results [32,33]. Beyond all these considerations, the whistleblower speeches relayed by some interviewees about the poor reception of modern contraceptive services suggested creating a welcoming environment for women. This analysis presented a resemblance to that of Dehlendorf *et al*. [34], for whom health workers must be welcoming during their counselling to put patients at ease.

This study has several limitations: 1) the subjectivity of the investigator could influence the results during the data analysis. However, she adopted a neutral perspective; 2) the researcher herself served as a health worker in the urban municipality of Dori may have influenced the participants' answers in one way or another. The use of triangulation and the verification of results helped reinforce the results' confirmability and reliability; 3) as will all qualitative research, the results of this study are only transferable to similar contexts. As the Sahel region has four urban municipalities, the study could have included all of them.

## Conclusion

The use of modern contraceptive methods is not encouraged by religious leaders despite the importance of birth spacing. For them, these methods run counter to their religious faith. They, therefore, prefer natural contraception and abstinence. Given the high level of influence of religious leaders in the socio-political landscape of Burkina Faso, interventions that engage religious of different faiths are crucial to increase the use of modern contraceptives in the country. By sharing specific biblical messages that address essential information about reproductive health and positive behaviour changes, religious leaders create an enabling environment for women and their partners to make healthy family planning decisions for themselves and their families.

### What is known about this topic

- The rate of unmet need for family planning is still high in sub-Saharan African countries and Burkina Faso;
- The low involvement of religious leaders and the still high fertility preferences of women of childbearing age, especially in rural areas in sub-Saharan Africa, are the main factors behind the low contraceptive prevalence in these countries.

### What this study adds

- Religious leaders of different faiths are advocacy resource persons who perpetuate norms and influence family planning and modern contraceptive use;
- It is necessary to strengthen and extend the dialogue with religious denominations down to the health centre level;
- Future studies are needed to accentuate further the views of religious leaders for their engagement with rural providers.

## Annex

**Annex 1**: consolidated criteria for reporting qualitative studies (COREQ): 32-item checklist
